# Influence of geography and environment on patterns of genetic differentiation in a widespread submerged macrophyte, Eurasian watermilfoil (*Myriophyllum spicatum* L., Haloragaceae)

**DOI:** 10.1002/ece3.1882

**Published:** 2016-01-08

**Authors:** Zhigang Wu, Dan Yu, Xing Li, Xinwei Xu

**Affiliations:** ^1^ National Field Station of Freshwater Ecosystem of Liangzi LakeCollege of Life SciencesWuhan UniversityWuhanChina

**Keywords:** Aquatic plant, China, geographic barrier, isolation by environment, landscape genetic, *Myriophyllum spicatum*

## Abstract

The effects of geographic and environmental variables on the pattern of genetic differentiation have been thoroughly studied, whereas empirical studies on aquatic plants are rare. We examined the spatial genetic differentiation of 58 *Myriophyllum spicatum* populations distributed throughout China with 12 microsatellite loci, and we analyzed its association with geographic distance, geographic barriers, and environmental dissimilarity using causal modeling and multiple matrix regression with randomization (MMRR) analysis. Two genetic clusters were identified, and their geographic distribution suggested mountain ranges as a barrier to gene flow. The causal modeling revealed that both climate and geographic barriers significantly influenced genetic divergence among *M. spicatum* populations and that climate had the highest regression coefficient according to the MMRR analysis. This study showed that geography and environment together played roles in shaping the genetic structure of *M. spicatum* and that the influence of environment was greater. Our findings emphasized the potential importance of the environment in producing population genetic differentiation in aquatic plants at a large geographic scale.

## Introduction

Assessing the relative importance of environmental and geographic factors on the genetic structure of populations is one of the fundamental issues of population genetic studies. Isolation by distance (IBD) is the common model of gene flow and often serves as an explanation for the effect of geographic isolation on the pattern of genetic differentiation (Slatkin 1987; Jenkins et al. 2010; Lee and Mitchell‐Olds 2011). Recently, the influences of the geographic factors besides distance and ecological selection on population genetic differentiation have been well discussed (Cushman et al. 2006; McRae 2006; Storfer et al. 2010; Lee and Mitchell‐Olds 2011; Shafer and Wolf 2013; Wang and Bradburd 2014). The heterogeneity of habitat and environment among populations may influence gene flow via disrupting or biased dispersal as well as selection against immigrants or interlineage hybrids, resulting in a pattern of isolation by environment (IBE) in which genetic differentiation is positively correlated with environmental dissimilarity (Wang and Summers 2010; Wang and Bradburd 2014).

Both patterns of IBD and IBE are often present simultaneously in nature (Sexton et al. 2014). Dissection of the relative importance of geography and environment on shaping patterns of genetic variation can help us obtain a deeper understanding of how landscape factors influence evolutionary processes (Wang and Bradburd 2014). By combining landscape and environmental data with novel analytical approaches, many relevant studies focusing on this theme have emerged (Storfer et al. 2007; Kozak et al. 2008; Wang 2013; Wang et al. 2013). However, relatively few empirical studies have examined the geographic and ecological roles in shaping genetic patterns of plant populations (Storfer et al. 2010), especially for aquatic plants in which different evolutionary processes are present (Barrett et al. 1993). Aquatic plants live in aquatic habitats representing geographic islands in terrestrial landscapes. Understanding the environmental drivers of genetic differentiation at a large geographic scale is important for species that have an island‐like distribution.

Eurasian watermilfoil (*Myriophyllum spicatum* L.) is a widespread submerged aquatic plant that is native to Europe, Asia, and northern Africa (Couch and Nelson 1985). This species occurs in various types of inland water bodies as it is tolerant to a wide range of water conditions (Aiken et al. 1979; Madsen 1998; Buchan and Padilla 2000). This species is hexaploid and presents a mixed reproduction system propagating through both sexual seeds and asexual propagules (stolons and plant fragments) (Aiken et al. 1979). Eurasian watermilfoil was likely introduced to North America in the 1940s (Couch and Nelson 1985) and has been recognized as a noxious weed due to its ability to rapidly spread in new habitats (Reed 1977). Eurasian watermilfoil is concerned greatly in North America because of its high invasiveness; however, it has been little studied in its native range.

In China, Eurasian watermilfoil is present throughout the whole country, including the Qinghai‐Tibetan Plateau (QTP) region (Yu et al. 2002). In this study, we sampled a total of 58 populations that approximately covered the distribution range of *M. spicatum* in China for population genetic analysis with microsatellite markers. Multiple approaches were used to detect the multivariate relationships (geographic and environmental variables) in the spatial analysis of genetic data. We aimed to assess the influence of geography and environment on the pattern of gene flow and genetic differentiation in Eurasian watermilfoil populations in China.

## Materials and Methods

### Sample collection

A total of 869 individuals of *M. spicatum* were collected at 58 sites throughout China, including the QTP region, during the summers of 2011 and 2012 (Appendix S1). Eight to 20 individuals from each population were sampled according to the size of habitat occupied by each population. The plants were collected randomly at intervals of at least 10 m to avoid collecting ramets from a single genet in the clonal Eurasian watermilfoil. Voucher specimens were deposited in the herbarium of Wuhan University (WH). A closely related species, *M. sibiricum*, co‐occurs in the QTP region and northeast China (Yu et al. 2002), and hybridization between *M. spicatum* and *M. sibiricum* has been reported in North America (Moody and Les 2002, 2007). In our previous study, we confirmed that hybridization between these two species also occurred in China (Wu et al. 2015). Hybrid populations identified by morphological traits and genetic data were not included in this study.

### Genetic data and statistical analysis

Fresh leaves were dried in silica gel in the field and frozen at −20°C after being transported to the laboratory. Total genomic DNA was extracted using the DNA Secure Plant Kit (Tiangen Biotech, Beijing, China) following the manufacturer's protocol.

We previously isolated 20 microsatellite loci from *M. spicatum* (Wu et al. 2013). Twelve of these loci (Myrsp1‐2, Myrsp4‐6, Myrsp9, Myrsp12‐16, and Myrsp18) were used in this study due to their high levels of polymorphism. The protocols for polymerase chain reaction (PCR) amplification and the analyses of the obtained PCR products followed Wu et al. (2013). Genotyping was performed using GeneMapper 4.0 software (Applied Biosystems, Foster City, CA).

For each population, as indices of genetic diversity, the number of alleles (NA), the effective number of alleles (ENA), Nei's (1987) diversity index (corrected for sample size) (D), and the number of genotypes were calculated using GENOTYPE and GENODIVE (Meirmans and Van Tienderen 2004). Clone assignment was also conducted by GENOTYPE and GENODIVE with the criterion of treating individuals with the same multilocus genotype as a clone (genet), and only the genotypes of genets were kept for subsequent analyses.

Because the allelic copies of microsatellites are ambiguous in polyploid species, we could not determine the exact genotypes of heterozygotes in *M. spicatum* due to dosage problems. Additionally, the statistics of microsatellite markers were developed for diploid organisms and not suitable for polyploid organisms (Bruvo et al. 2004). Therefore, we converted the microsatellite data to a binary format as dominant data (Lynch 1990; Samadi et al. 1999) in R software version 3.1.1 (R Development Core Team, 2014) with the POLYSAT package (Clark and Jasieniuk 2011). *Φ*_PT_ from the analysis of molecular variance, which is analogous to Nei's *F*_ST_, was used as a measure of population genetic differentiation for binary data (Peakall et al. 1995; Maguire et al. 2002), and the pairwise *Φ*_PT_ among populations was calculated in GenAlEx 6.5 (Peakall and Smouse 2012). To estimate the influence of small sample size, we used 100 replicates of *Φ*_PT_ calculation based on a random sampling of six individuals from populations with more than 10 individuals (genets) (Ortego et al. 2015). Comparable and highly correlated *Φ*_PT_ estimates across different replicates (Mantel *r *=* *0.859 ± 0.117, all *P* values < 0.001) were obtained indicating that a sample size of no less than six can provide reliable evaluation of pairwise *Φ*_PT_‐values. Therefore, a total of 43 populations containing no less than six genets were included in the analysis of genetic differentiation.

Genetically distinct clusters of the 43 Eurasian watermilfoil populations were identified using an individual‐based assignment approach as implemented in STRUCTURE 2.3.4 (Pritchard et al. 2000; Falush et al. 2003). The current version of this software can accommodate dominant markers (Falush et al. 2007). Twenty independent runs were performed for each *K* value (*K* = 1–20) with a burn‐in period of 20,000 iterations and 100,000 MCMC iterations under the admixture model. The best‐fit number of clusters was determined based on the ∆*K* method (Evanno et al. 2005). Discriminant analysis of principal components (DAPC; Jombart et al. 2010) was also used as an alternative method for inferring patterns of the genetic structure. The method is a multivariate approach combining principal component analysis and discriminant analysis, which required priori clustering algorithms determined by *k*‐means. DAPC is an adequate clustering analysis for polyploids and does not require populations in Hardy–Weinberg equilibrium (Dufresne et al. 2014). We evaluated up to *k* = 20 groups, and Bayesian information criterion (BIC) was used to assess the number of clusters best fitting the data (Jombart et al. 2010). However, the value of BIC decreased with the increase in *k*. We therefore set an identical *k* value as the K of STRUCTURE for comparison. DAPC was implemented in R with “adegenet” version 1.4‐1 (Jombart 2008).

### Landscape genetic analysis

Environment variables were extracted (to a resolution of 1 km) for the studied sites from the BioClim layers (Busby 1991; Hijmans et al. 2005) using ArcGIS version 10.0 (Environmental Systems Research Institute, Redlands, CA). We reduced the environmental variables by the components of principal component analysis (PCA) based on the 19 BioClim variables using the “prcomp” function in R. The PCA scores of the 43 studied sites in the environmental space and environmental (Euclidean) distances between populations were calculated based on values of the first two PCA axes. Both environmental and geographic distances were calculated using PASSaGE 2 software (Rosenberg and Anderson 2011). To quantify the isolating effect of barriers identified by spatial genetic structure (the linear arrangement of the mountain ranges: Daxing'anling Mountains, Taihang Mountains, Qinling Mountains, and Hengduan Mountains; see Results) on gene flow, a categorical matrix was constructed using a binary assignment with 0 for populations from one side and 1 for those located on the opposite side of the barrier (Cushman et al. 2006; Robertson et al. 2009; Legendre and Legendre 2012).

The correlations between genetic differentiation and geographic/environmental factors were determined by a combination of Mantel tests and matrix regression analysis with a distance matrix. We performed simple Mantel tests for the correlation of genetic differentiation with geographic distance, barrier matrix, and environmental distance. When all of the three factors were significantly correlated to genetic differentiation (see Results), partial Mantel tests (Smouse et al. 1986) between genetic differentiation and one factor under the influence of the other two factors (as covariates) were further performed with 10,000 permutations (causal modeling; Legendre 1993; Cushman et al. 2006; Appendix S2). Both simple and partial Mantel tests were conducted in R using the “ecodist” package (Goslee and Urban 2007). In addition, the multiple matrix regression with randomization (MMRR) was used to estimate the independent effect of the factors, which has been shown to be robust toward a wide range of dispersal rates (Wang 2013). The analysis was implemented with 10,000 permutations in R with the MMRR function script (Wang 2013).

## Results

### Genetic analysis

The number of alleles (NA), the effective number of alleles (ENA), and genotype diversity (D) ranged from 22 to 84, 22 to 59.184, and 0 to 1 with a mean of 51.276, 42.054, and 0.756, respectively (Appendix S1). Five populations, namely XJ4, SHX, SX1, NM1, and LN4, were genetically monomorphic indicating that all samples were clones of a single genet within each population. A total of 496 genotypes were revealed in the 869 samples, and nearly all of the obtained genotypes were population‐specific with the exception of two genotypes shared by GX1 and GX2 as well as four genotypes shared by HEB2 and TJ (see multilocus genotype data in Dryad for details). The overall genetic differentiation (*Φ*_PT_) was 0.521 with the maximum of 0.907 between TB5 and GD and the minimum of 0.141 between JS and SD3. All pairwise *Φ*_PT_ were significant (*P *<* *0.05).

**Figure 1:**
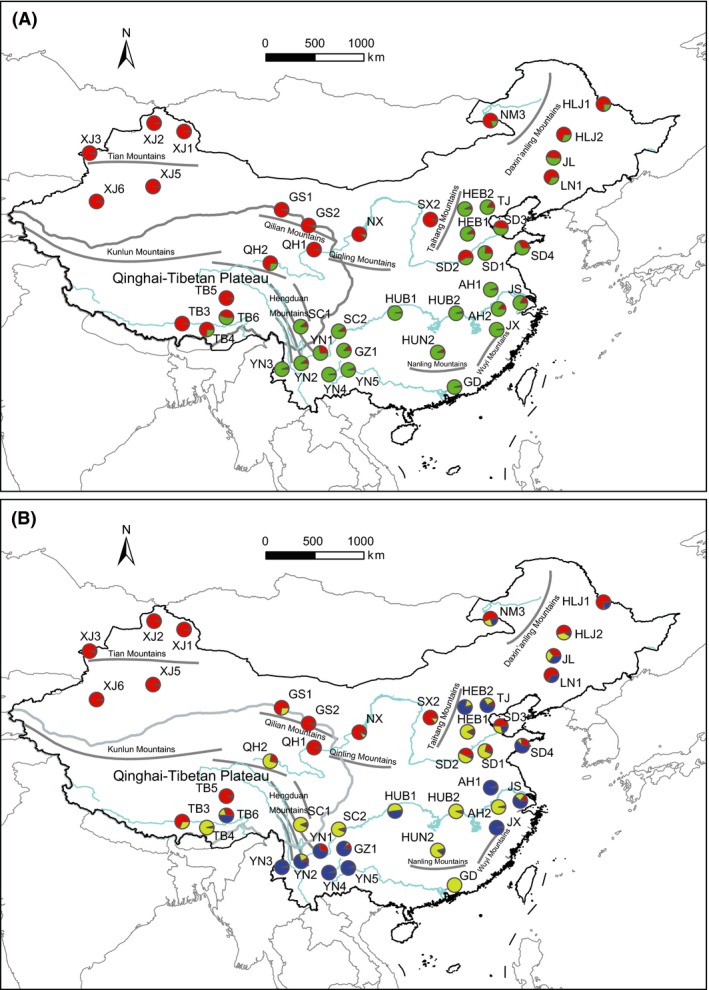
Sampling sites for *Myriophyllum spicatum* in China. Pie charts represent the probability of assignment to the subclusters when *K* = 2 (A) and *K* = 3 (B) as identified by STRUCTURE based on microsatellite data. Population codes are shown on the side. The main mountain ranges and rivers of China as well as the Qinghai‐Tibetan Plateau region are outlined.

The STRUCTURE analysis suggested *K* = 2 as the optimal number of clusters based on the value of ∆*K* (Appendix S3). Cluster 1 (northwestern cluster) comprised mainly populations from the northern and western part of China, including northeast China, northwest China, and the QTP region, while Cluster 2 (southeastern cluster) was distributed across the southern and eastern part of China (Fig. 1A). The result of *K* = 3 was also presented due to the high value of ∆*K* (Appendix S3). When *K* = 3, the northwestern cluster was retained with the exception of two populations (QH2 and TB4), while the southeastern cluster was subdivided into two subclusters with no obvious intracluster genetic structure (Fig. 1B). Results of the DAPC analysis were analogous to those of the STRUCTURE analysis except for a few populations (GS1, QH2, TB4, YN1, HUB1) with different proportion of genetic clusters (Appendix S4).

### Landscape genetic analysis

The first two PCA axes explained 61.9% of the variance in the climatic variables used for the quantification of environmental space. The first axis was closely associated with temperature, and the second axis was associated with precipitation (Appendix S5).

**Figure 2:**
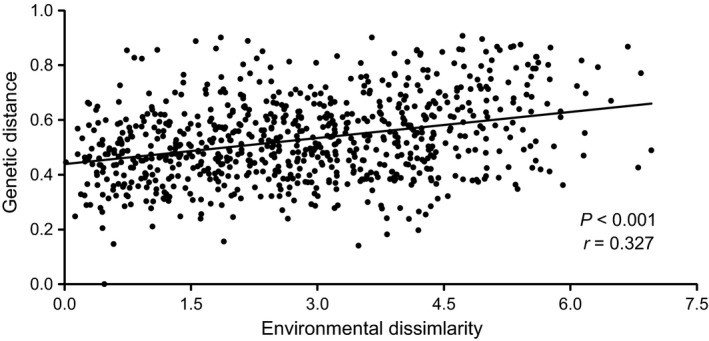
Scatter plots of Mantel tests for correlation between genetic differentiation and environmental dissimilarity in *Myriophyllum spicatum*.

Significantly positive correlations were found between genetic and geographic distances, between genetic differentiation and barrier matrices, and between genetic and environmental distances (Table 1, Fig. 2). When the influence of the other two factors were controlled, the genotype–environment and genotype–barrier associations remained significant, whereas no significant correlations existed between genetic differentiation and geographic distance (Table 1). According to the MMRR analysis, the climate had the highest regression coefficient, while the effects of geographic factors were not significant (Table 2).

**Table 1:** Simple and partial Mantel tests of association between genetic distance and geographic distance, geographic barrier, and environmental dissimilarity of *Myriophyllum spicatum* populations

Landscape feature	Controlled	*r*	*P*
Dist		0.210	**0.017**
Barrier		0.192	**0.001**
Env		0.327	**0.001**
Dist	Barrier	0.147	0.105
Dist	Env	0.085	0.384
Barrier	Dist	0.120	**0.048**
Barrier	Env	0.141	**0.039**
Env	Dist	0.269	**0.004**
Env	Barrier	0.302	**0.003**

Dist, geographic distance; Barrier, categorical matrix of geographic barrier; Env, environmental dissimilarity. Significant values are presented in bold.

**Table 2:** Regression coefficient (*β*) and significance (*P*) of MMRR analysis on the association between genetic distance and geographic distance, geographic barrier, and environmental dissimilarity of *Myriophyllum spicatum* populations in China

Landscape feature	*β*	*P*
Geographic distance	0.063	0.541
Geographic barrier	0.093	0.216
Environmental dissimilarity	0.310	**0.004**

Significant values are presented in bold.

## Discussion

It is generally accepted that IBD is one of the main factors that drive genetic divergence of natural populations. For aquatic plants, habitat fragmentation is severe because they live in the discrete wetlands in terrestrial landscapes, which may reduce gene flow and strengthen the isolation (Young et al. 1996). Many studies have revealed strong interpopulation genetic divergence and patterns of IBD in aquatic plants (Gornall et al. 1998; King et al. 2002; Nies and Reusch 2005; reviewed in Barrett et al. 1993; Santamaría 2002). Recently, landscape genetic studies have suggested that geography is merely one of many important factors influencing gene flow and evolutionary processes, and other landscape elements (e.g., environmental heterogeneity) might be decisive as well (Nosil et al. 2005; Lee and Mitchell‐Olds 2011; Wang and Bradburd 2014). However, few empirical studies have examined the roles of environmental factors in shaping genetic patterns of aquatic plant populations (Storfer et al. 2010; but see Zhou et al. 2013). Given the autocorrelation between geography and several ecological factors (e.g., climate), it is quite a challenge to disentangle the relative roles of geography and ecology in observed patterns of spatial genetic differentiation in a landscape. The MMRR, a newly developed modeling and statistical method, has been proved to be robust to simultaneously quantify the effects of multiple factors on a single response variable (e.g., genetic distance), especially at low‐to‐moderate dispersal levels and when the effects of the contributors were not highly differentiated (*β*_1_/*β*_2_ ≤ 5, Wang 2013). In this study, using the MMRR and partial Mantel tests, we revealed greater influences of environment (climate) and geographic barriers than geographic distance on the genetic divergence among *M. spicatum* populations (Tables 1 and 2).

Nearly all of the obtained genotypes that were population‐specific suggested a high rate of genetic recombination and uncommon interpopulation dispersal of vegetative propagules in *M. spicatum*. For aquatic plants, the isolation of independent basins constituted a barrier to interbasin gene flow. However, it is possible that animal activity, for example, waterbird migration, could transport viable propagules across water basins (Clausen et al. 2002; Figuerola and Green 2002; Santamaría 2002). The fact that some waterbirds (such as *Anas crecca*) feed on the seeds of *Myriophyllum* (Olney 1963) and the seed is viable after passing through the bird's digestive track (Brochet et al. 2010) indicates that interbasin dispersal of seeds was likely in the Eurasian watermilfoil. No genetic subclusters corresponding to geographic ranges were found in the southeastern cluster (Fig. 1B, Appendix S4) indicating that dispersals of seeds across river basins might be frequent. In China, the waterfowl (including *A. crecca*) mainly breed north of China and migrate southward for the winter, and the waterfowl rarely travel from west to east or in the opposite direction (Sibley and Monroe 1990; MacKinnon et al. 1996). Similarly, several empirical studies on the transmission of avian influenza virus have also suggested that the waterfowl might not travel between western and eastern lakes in China (Takekawa et al. 2010; Prosser et al. 2011). The inferred migration routes of the waterfowl were mainly coincident with the geographic distribution of two genetic clusters and barrier effect of several main mountain ranges (Taihang Mountains, Qinling Mountains, and Hengduan Mountains) for Eurasian watermilfoil populations. Taken together, limited dispersal events between northwestern and southeastern regions (across mountain ranges) but frequent dispersal events within the two regions via migratory waterfowl might help to explain the spatial genetic structure of *M. spicatum* in China.

An obvious IBE pattern was revealed in *M. spicatum* (Fig. 2, Tables 1 and 2), suggesting a significant effect of environmental factors on the effective dispersal among populations. Selection against immigrants or reduced fitness of interlineage hybrids are common processes to generate IBE, which implied local adaptation to heterogeneous environments (Pflüger and Balkenhol 2014; Wang and Bradburd 2014). Many species with IBE have shown local adaptation to environmental gradients, and more importantly, a reduction in gene flow among populations in heterogeneous environments and the reinforcement of local adaptation due to IBE (Mitchell‐Olds et al. 2007; Byars et al. 2009; Barker et al. 2011; Nosil 2012; Shafer and Wolf 2013; Sexton et al. 2014). The climate variables, especially temperature, are important abiotic factors affecting the performance and distribution of aquatic plants (Lacoul and Freedman 2006; Bornette and Puijalon 2011). The first PCA axis closely associated with temperature (Appendix S5) also indicated that temperature was an important factor related to the differentiation and potential adaptation of *M. spicatum* populations. The two genetic clusters of *M. spicatum* identified here occupied different climate conditions. The northern and QTP populations live in temperate zone and alpine region, which is colder than subtropical zone where the eastern and southern populations live. The climate can influence *M. spicatum* through life history traits, tolerance of young seedlings, and temperature requirement for initial growth (Patten 1956; Aiken et al. 1979; Nichols and Shaw 1986; Smith and Barko 1990; Xiao et al. 2010). These examples imply a potential of local adaptation to different climatic conditions in *M. spicatum*. Although more studies (e.g., common garden) are needed to confirm the contributions of environmental factors to the pattern observed in *M. spicatum*, our study suggests the important role of ecological factors in the formation and maintenance of genetic differentiation in *M. spicatum*.

In conclusion, our study revealed both geographic barrier and climate could influence the pattern of gene flow in Eurasian watermilfoil populations and a potentially greater prediction of IBE on the genetic differentiation of *M. spicatum*. As one of several case studies, our findings suggest the importance of climate in shaping patterns of genetic variation and imply a potential of local adaptation to different climatic conditions in aquatic plants at a large geographic scale.

## Conflict of Interest

None declared.

## Data Accessibility

The microsatellite genotype data and the BioClim variables of the studied samples were deposited in Dryad (http://dx.doi.org/10.5061/dryad.9v2g5).

## Supporting information


**Appendix S1.** Geographic origins, voucher information, habitat types, sample sizes (*n*), number of genets (Ng), number of alleles (NA), effective number of alleles (ENA) and Nei's genotypic diversity index (corrected for sample size) (D) for the 58 *Myriophyllum spicatum* populations.

**Appendix S2.** Causal model of the expected relationship between genetic distances and geographic/environmental variables.

**Appendix S3.** Modelling of the number of genetic clusters in *Myriophyllum spicatum* using STRUCTURE.

**Appendix S4.** Spatial genetic structure using DAPC analysis.

**Appendix S5.** Weighting of each climatic variable in the PCA analyses.
