# Supplementary material for: Influence of geography and environment on patterns of genetic differentiation in a widespread submerged macrophyte, Eurasian watermilfoil (Myriophyllum spicatum L., Haloragaceae)
Source: Ecol Evol. 2016 Jan 8;6(2):460–8. doi: 10.1002/ece3.1882 (PMC4729246; doi:10.1002/ece3.1882)
Supplement: Supplementary file 1 — Appendix S1. Geographic origins, voucher information, habitat types, sample sizes (n), number of genets (Ng), number of alleles (NA), effective number of alleles (ENA) and Nei's genotypic diversity index (corrected for sample size) (D) for the 58 Myriophyllum spicatum populations. [file ECE3-6-460-s001.docx]

**Appendix 1:** Geographic origins, voucher information, habitat types, sample sizes (n), number of genets (Ng), number of alleles (NA), effective number of alleles (ENA) and Nei's genotypic diversity index (corrected for sample size) (D) for the 58 *Myriophyllum spicatum* populations. All voucher specimens are deposited at the Wuhan University Herbarium (WH).

| Population | Location | Voucher no. | Coordinates | Altitude | Habitat | n | Ng | NA | ENA | D |
| --- | --- | --- | --- | --- | --- | --- | --- | --- | --- | --- |
| XJ1 | Fuyun, Xijiang | Xu et al., 4157 | 46.9644N^o^,89.5815E^o^ | 876 | Wetland | 18 | 18 | 38 | 34.092 | 1 |
| XJ2 | Buerjin, Xijiang | Xu et al., 2543 | 47.7296N^o^,86.8201E^o^ | 466 | River | 20 | 15 | 29 | 25.336 | 0.968 |
| XJ3 | Wenquan, Xijiang | Xu et al., 4250 | 44.9813N^o^,80.9058E^o^ | 1457 | Wetland | 16 | 7 | 45 | 35.748 | 0.692 |
| XJ4 | Huocheng, Xijiang | Xu et al., 2556 | 43.9442N^o^,80.8715E^o^ | 546 | River | 18 | 1 | 22 | 22 | 0 |
| XJ5 | Bohu, Xijiang | Xu et al., 2570 | 41.9068N^o^,86.7315E^o^ | 1020 | Lake | 20 | 7 | 32 | 27.444 | 0.584 |
| XJ6 | Alaer, Xijiang | Xu et al., 4409 | 40.5415N^o^,81.5489E^o^ | 1005 | Wetland | 14 | 8 | 46 | 39.876 | 0.923 |
| GS1 | Jiuquan, Gansu | Xu et al., 2529 | 39.7715N^o^,98.5421E^o^ | 1350 | Wetland | 18 | 9 | 42 | 38.112 | 0.895 |
| GS2 | Yongchang, Gansu | Xu et al., 2519 | 38.323N^o^,101.0083E^o^ | 1890 | Wetland | 20 | 13 | 49 | 40.512 | 0.911 |
| QH1 | Guide, Qinghai | Xu et al., 5076 | 36.1117N^o^,101.5162E^o^ | 2194 | Wetland | 18 | 12 | 48 | 40.332 | 0.922 |
| QH2 | Maduo, Qinghai | Xu et al., 5087 | 34.8619N^o^,97.4919E^o^ | 4274 | Lake | 18 | 15 | 55 | 47.424 | 0.974 |
| TB1 | Zhongba, Tibet | Xu et al., 5130 | 29.6957N^o^,84.1284E^o^ | 4569 | River | 14 | 2 | 37 | 36.276 | 0.264 |
| TB2 | Xainza, Tibet | Xu et al., 5102 | 30.7542N^o^,88.7873E^o^ | 4692 | Wetland | 18 | 2 | 35 | 33.216 | 0.111 |
| TB3 | Shigatse, Tibet | Xu et al., 5167 | 29.3245N^o^,89.4102E^o^ | 3787 | Wetland | 18 | 10 | 48 | 41.832 | 0.876 |
| TB4 | Comai, Tibet | Xu et al., 5176 | 28.771N^o^,91.6686E^o^ | 4626 | Lake | 18 | 6 | 39 | 36.168 | 0.81 |
| TB5 | Gongbo'gvamda, Tibet | Xu et al., 2472 | 29.8927N^o^,93.4645E^o^ | 3560 | Wetland | 20 | 9 | 32 | 29.484 | 0.879 |
| TB6 | Nyingchi, Tibet | Xu et al., 2478 | 29.6309N^o^,94.3825E^o^ | 2990 | Wetland | 12 | 12 | 57 | 34.335 | 1 |
| YN1 | Yongsheng, Yunnan | Xu et al., 2316 | 26.6237N^o^,100.6526E^o^ | 1520 | Lake | 20 | 8 | 46 | 42.72 | 0.805 |
| YN2 | Dali, Yunnan | Xu et al., 2277 | 25.6708N^o^,100.2109E^o^ | 1954 | Lake | 19 | 18 | 74 | 51.252 | 0.994 |
| YN3 | Tengchong, Yunnan | Xu et al., 2256 | 25.1282N^o^,98.5604E^o^ | 1719 | Lake | 17 | 12 | 63 | 46.212 | 0.934 |
| YN4 | Chengjiang, Yunnan | Xu et al., 2206 | 24.6323N^o^,102.8828E^o^ | 1735 | Lake | 20 | 16 | 67 | 48.648 | 0.979 |
| YN5 | Xuanwei, Yunnan | Xu et al., 2192 | 26.2498N^o^,104.0621E^o^ | 2087 | Wetland | 20 | 6 | 52 | 46.092 | 0.779 |
| SC1 | Daocheng, Sichuan | Xu et al., 5511 | 29.0368N^o^,100.2985E^o^ | 3776 | Wetland | 12 | 8 | 84 | 57.42 | 0.894 |
| SC2 | Leibo, Sichuan | Xu et al., 2427 | 28.4286N^o^,103.7854E^o^ | 1134 | Lake | 16 | 6 | 48 | 39.828 | 0.542 |
| GZ1 | Weining, Guizhou | Xu et al., 2178 | 26.8383N^o^,104.2645E^o^ | 2187 | Lake | 13 | 13 | 67 | 47.94 | 1 |
| GZ2 | Guiding, Guizhou | Xu et al., 2144 | 25.3743N^o^,107.444E^o^ | 956 | Wetland | 13 | 4 | 47 | 42.408 | 0.423 |
| GX1 | Yongfu, Guangxi | Xu et al., 2062 | 25.0205N^o^,109.9759E^o^ | 143 | River | 12 | 5 | 66 | 45.48 | 0.576 |
| GX2 | Lipu, Guangxi | Xu et al., 2128 | 24.4894N^o^,110.3951E^o^ | 221 | River | 17 | 3 | 43 | 41.244 | 0.522 |
| GD | Boluo, Guangdong | Xu et al., 3323 | 23.4936N^o^,114.4079E^o^ | 70 | River | 10 | 9 | 46 | 43.128 | 0.978 |
| HUN1 | Linxiang, Hunan | Xu et al., 2072 | 29.3389N^o^,113.3962E^o^ | 87 | River | 10 | 3 | 43 | 40.284 | 0.711 |
| HUN2 | Hengnan, Hunan | Xu et al., 2094 | 26.6696N^o^,112.8615E^o^ | 50 | Wetland | 14 | 7 | 59 | 49.152 | 0.692 |
| JX | Yushan, Jiangxi | Xu et al., 3423 | 28.7558N^o^,118.2881E^o^ | 145 | River | 18 | 10 | 44 | 39.516 | 0.902 |
| HUB1 | Lichuan, Hubei | Xu et al., 2436 | 30.2947N^o^,108.9514E^o^ | 1102 | River | 17 | 9 | 48 | 40.452 | 0.875 |
| HUB2 | Wuhan, Hubei | Xu et al., 2616 | 30.2653N^o^,114.5578E^o^ | 21 | Lake | 19 | 16 | 73 | 50.772 | 0.982 |
| JS | Suzhou, Jiangsu | Xu et al., 1017 | 31.2227N^o^,120.4461E^o^ | 0 | Lake | 12 | 11 | 84 | 59.184 | 0.985 |
| AH1 | Hefei, Anhui | Xu et al., 5269 | 31.5315N^o^,117.7423E^o^ | 65 | Lake | 13 | 8 | 58 | 49.044 | 0.859 |
| AH2 | Jingxian, Anhui | Xu et al., 5295 | 30.6623N^o^,118.4499E^o^ | 78 | Wetland | 14 | 11 | 47 | 41.268 | 0.956 |
| HEN | Anyang, Henan | Xu et al., 3563 | 36.0415N^o^,114.1155E^o^ | 138 | River | 8 | 4 | 42 | 36.672 | 0.75 |
| SD1 | Dongping, Shandong | Xu et al., 3607 | 35.9037N^o^,116.2282E^o^ | 86 | Lake | 19 | 18 | 72 | 47.052 | 0.994 |
| SD2 | Heze, Shandong | Xu et al., 3589 | 35.3913N^o^,115.4529E^o^ | 60 | Wetland | 14 | 6 | 57 | 41.244 | 0.681 |
| SD3 | Dongying, Shandong | Xu et al., 6282 | 38.1026N^o^,118.6747E^o^ | 1 | Wetland | 10 | 10 | 67 | 51.9 | 1 |
| SD4 | Qingdao, Shandong | Xu et al., 6315 | 36.2927N^o^,120.6233E^o^ | 15 | Wetland | 8 | 8 | 59 | 50.712 | 1 |
| HEB1 | Jizhou, Hebei | Xu et al., 3672 | 37.5711N^o^,115.6095E^o^ | 38 | Lake | 17 | 17 | 63 | 48.804 | 1 |
| HEB2 | Laishui, Hebei | Xu et al., 2608 | 39.8395N^o^,115.3664E^o^ | 424 | Wetland | 19 | 10 | 65 | 50.424 | 0.883 |
| TJ | Jixian, TJ | Xu et al., 2612 | 40.0213N^o^,117.4354E^o^ | 20 | Wetland | 20 | 14 | 70 | 52.596 | 0.889 |
| SHX | Baoji, Shanxi | Xu et al., 2601 | 34.3375N^o^,107.3717E^o^ | 540 | River | 9 | 1 | 38 | 37.236 | 0 |
| NX | Zhongwei, Ningxia | Xu et al., 6583 | 37.523N^o^,105.6741E^o^ | 1183 | Wetland | 14 | 10 | 53 | 43.128 | 0.945 |
| SX1 | Louxu, Shanxi | Xu et al., 2604 | 38.0335N^o^,111.9551E^o^ | 1065 | Wetland | 20 | 1 | 36 | 35.568 | 0 |
| SX2 | Ningwu, Shanxi | Xu et al., 5198 | 38.8726N^o^,112.2071E^o^ | 1669 | Lake | 18 | 18 | 57 | 42.24 | 1 |
| NM1 | Eji'naqi, Neimenggu | Xu et al., 6677 | 42.0093N^o^,101.5846E^o^ | 894 | Wetland | 9 | 1 | 35 | 34.668 | 0 |
| NM2 | Hangjinhouqi, Neimenggu | Xu et al., 6694 | 40.8456N^o^,107.2093E^o^ | 1035 | River | 8 | 3 | 39 | 33.816 | 0.464 |
| NM3 | Xinbaerhuyouqi, Neimenggu | Xu et al., 6223 | 47.9668N^o^,117.7166E^o^ | 580 | Lake | 18 | 14 | 64 | 47.22 | 0.954 |
| HLJ1 | Xunke, Heilongjiang | Xu et al., 3992 | 49.4731N^o^,128.0893E^o^ | 107 | Wetland | 9 | 7 | 54 | 42.156 | 0.917 |
| HLJ2 | Duerbote, Heilongjiang | Xu et al., 0682 | 46.6801N^o^,124.4597E^o^ | 215 | Lake | 10 | 8 | 65 | 50.04 | 0.956 |
| JL | Shuangliao, Jilin | Xu et al., 0728 | 43.7693N^o^,123.5174E^o^ | 231 | Wetland | 8 | 6 | 48 | 40.392 | 0.929 |
| LN1 | Kangping, Liaoning | Xu et al., 4644 | 42.7678N^o^,123.3167E^o^ | 6 | Lake | 9 | 9 | 66 | 50.364 | 1 |
| LN2 | Panshan, Liaoning | Xu et al., 3672 | 41.1707N^o^,121.7107E^o^ | 4 | Wetland | 10 | 4 | 49 | 44.784 | 0.733 |
| LN3 | Dawa, Liaoning | Xu et al., 1174 | 41.0704N^o^,121.9627E^o^ | 6 | Wetland | 14 | 3 | 38 | 36.408 | 0.538 |
| LN4 | Zhuanghe, Liaoning | Xu et al., 3737 | 39.8566N^o^,123.3968E^o^ | 5 | River | 12 | 1 | 38 | 37.464 | 0 |
| Overall |  |  |  |  |  | 869 | 496 | 146 | 39.926 | 0.995 |
| Mean |  |  |  |  |  |  |  | 51.276 | 42.054 | 0.756 |
