# Supplementary material for: Influence of geography and environment on patterns of genetic differentiation in a widespread submerged macrophyte, Eurasian watermilfoil (Myriophyllum spicatum L., Haloragaceae)
Source: Ecol Evol. 2016 Jan 8;6(2):460–8. doi: 10.1002/ece3.1882 (PMC4729246; doi:10.1002/ece3.1882)
Supplement: Supplementary file 2 — Appendix S2. Causal model of the expected relationship between genetic distances and geographic/environmental variables. [file ECE3-6-460-s002.docx]

**Appendix 2:** Causal model of the expected relationship between genetic distances and geographic/environmental variables.

| Model | Landscape feature | Controlled | Expected significance |
| --- | --- | --- | --- |
| Dist | Dist | Barrier | Sig |
|  | Dist | Env | Sig |
|  | Barrier | Dist | NS |
|  | Env | Dist | NS |
| Barrier | Barrier | Dist | Sig |
|  | Barrier | Env | Sig |
|  | Dist | Barrier | NS |
|  | Env | Barrier | NS |
| Env | Env | Dist | Sig |
|  | Env | Barrier | Sig |
|  | Dist | Env | NS |
|  | Barrier | Env | NS |
| Dist and Barrier | Dist | Barrier | Sig |
|  | Dist | Env | Sig |
|  | Barrier | Dist | Sig |
|  | Barrier | Env | Sig |
|  | Env | Dist | NS |
|  | Env | Barrier | NS |
| Dist and Env | Dist | Barrier | Sig |
|  | Dist | Env | Sig |
|  | Barrier | Dist | NS |
|  | Barrier | Env | NS |
|  | Env | Dist | Sig |
|  | Env | Barrier | Sig |
| Barrier and Env | Dist | Barrier | NS |
|  | Dist | Env | NS |
|  | Barrier | Dist | Sig |
|  | Barrier | Env | Sig |
|  | Env | Dist | Sig |
|  | Env | Barrier | Sig |
| Dist, Barrier and Env | Dist | Barrier | Sig |
|  | Dist | Env | Sig |
|  | Barrier | Dist | Sig |
|  | Barrier | Env | Sig |
|  | Env | Dist | Sig |
|  | Env | Barrier | Sig |

Dist, geographic distance; Barrier, categorical matrix of geographic barrier; Env, environmental distance. Sig, significant correlation between two matrices with the *p* < 0.05; NS, nonsignificant correlation with *p* > 0.05.
