# Supplementary material for: Influence of geography and environment on patterns of genetic differentiation in a widespread submerged macrophyte, Eurasian watermilfoil (Myriophyllum spicatum L., Haloragaceae)
Source: Ecol Evol. 2016 Jan 8;6(2):460–8. doi: 10.1002/ece3.1882 (PMC4729246; doi:10.1002/ece3.1882)

Appendix 3: Modelling of the number of genetic clusters in *Myriophyllum spicatum* using STRUCTURE. Delta K (ΔK) calculated based on Evanno *et al.* (2005), charted against the number of modeled genepools (K).

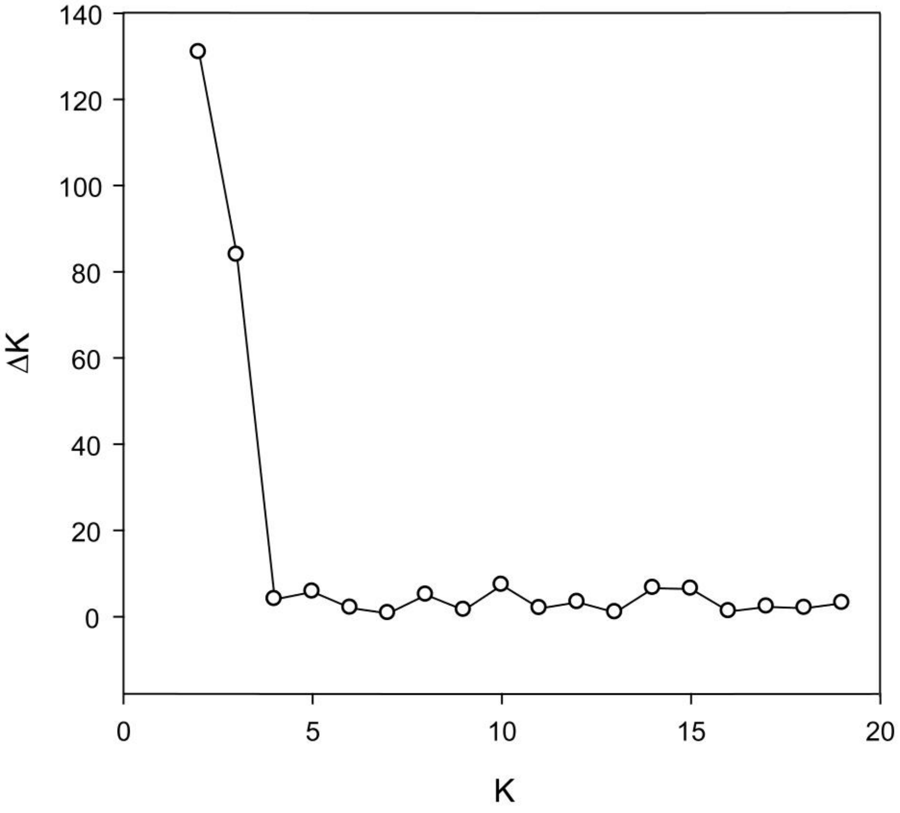

Supplement: Supplementary file 3 — Appendix S3. Modelling of the number of genetic clusters in Myriophyllum spicatum using STRUCTURE. [file ECE3-6-460-s003.pdf]
