# Supplementary material for: Influence of geography and environment on patterns of genetic differentiation in a widespread submerged macrophyte, Eurasian watermilfoil (Myriophyllum spicatum L., Haloragaceae)
Source: Ecol Evol. 2016 Jan 8;6(2):460–8. doi: 10.1002/ece3.1882 (PMC4729246; doi:10.1002/ece3.1882)

Appendix 4: Spatial genetic structure using DAPC analysis. Pie charts represent the proportion of the individuals assigned to the genetic clusters when k=2 (A) and k=3 (B) respectively. Population codes are shown on the side. The main mountain ranges and rivers of China, and the Qinghai-Tibetan Plateau region are outlined.

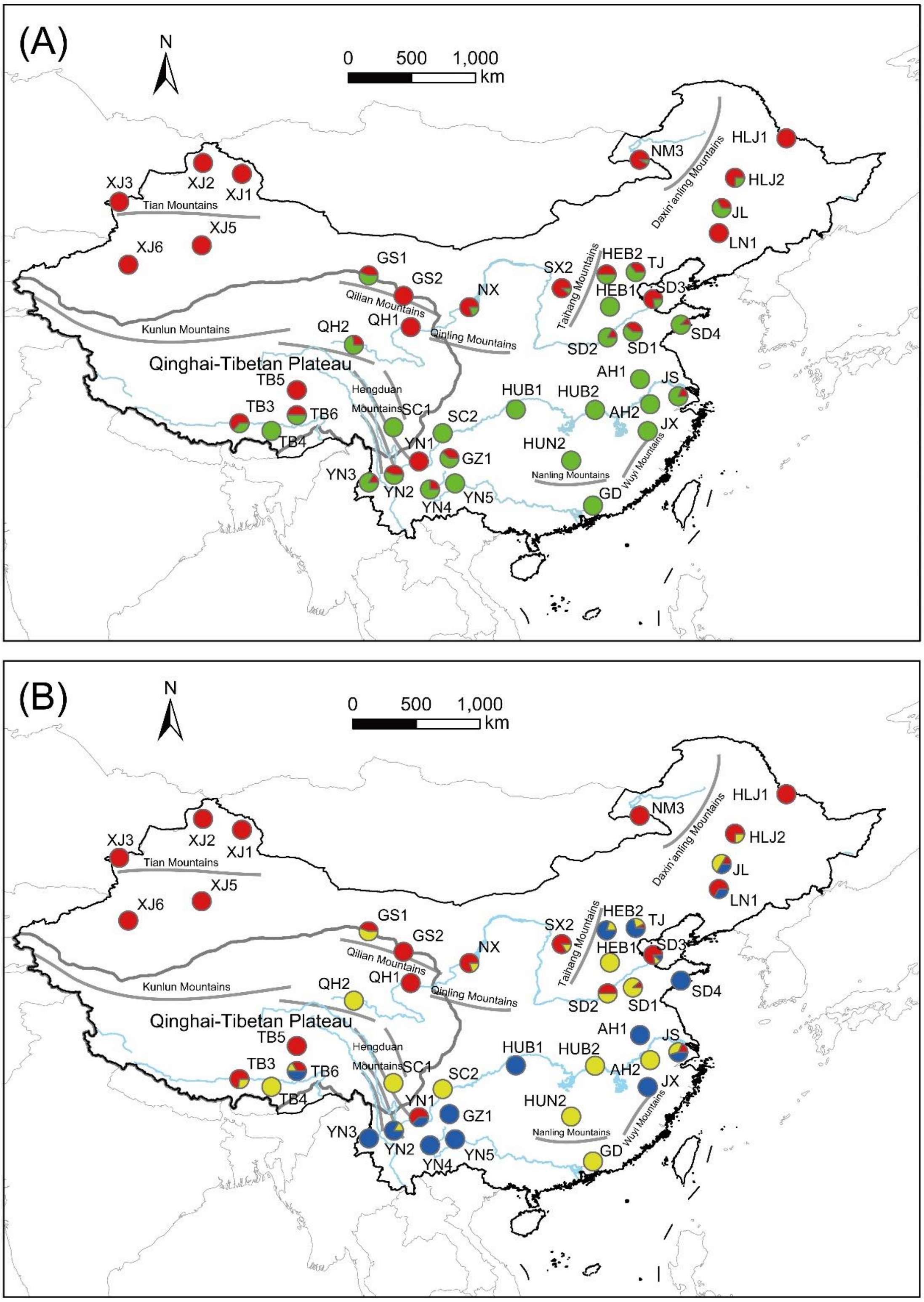

Supplement: Supplementary file 4 — Appendix S4. Spatial genetic structure using DAPC analysis. [file ECE3-6-460-s004.pdf]
