# Supplementary material for: Influence of geography and environment on patterns of genetic differentiation in a widespread submerged macrophyte, Eurasian watermilfoil (Myriophyllum spicatum L., Haloragaceae)
Source: Ecol Evol. 2016 Jan 8;6(2):460–8. doi: 10.1002/ece3.1882 (PMC4729246; doi:10.1002/ece3.1882)
Supplement: Supplementary file 5 — Appendix S5. Weighting of each climatic variable in the PCA analyses. [file ECE3-6-460-s005.docx]

**Appendix 5:** Weighting of each climatic variable in the PCA analyses. The values indicate the relative contributions of climatic variables to the first two axes.

|  | PC1 (49.0%) | PC2 (12.9%) |
| --- | --- | --- |
| BIO1 - Annual Mean Temperature | 0.165479 | -0.71208 |
| BIO2 - Mean of monthly (max temp - min temp) | -0.02505 | -0.02556 |
| BIO3 - Isothermality (BIO2/BIO7) * 100 | 0.007182 | -0.02897 |
| BIO4 - Temperature Seasonality (standard deviation *100) | -0.0359 | 0.01365 |
| BIO5 - Max Temperature of Warmest Month | 0.011807 | -0.03703 |
| BIO6 - Min Temperature of Coldeast Month | 0.424088 | 0.176349 |
| BIO7 - Temperature Annual Range (BIO5-BIO6) | -0.03187 | 0.002426 |
| BIO8 - Mean Temperature of Wettest Quarter | 0.020065 | -0.06983 |
| BIO9 - Mean Temperature of Driest Quarter | 0.589766 | -0.20994 |
| BIO10 - Mean Temperature of Warmest Quarter | 0.023814 | -0.05676 |
| BIO11 - Mean Temperature of Coldest Quarter | 0.590269 | -0.03861 |
| BIO12 - Annual Precipitation | 0.093702 | 0.205622 |
| BIO13 - Precipitation of Wettest Month | 0.070271 | 0.168923 |
| BIO14 - Precipitation of Driest Month | 0.133673 | 0.246083 |
| BIO15 - Precipitation Seasonality (Coefficient of Variation) | -0.02556 | -0.0444 |
| BIO16 - Precipitation of Wettest Quarter | 0.077294 | 0.177419 |
| BIO17 - Precipitation of Driest Quarter | 0.154065 | 0.318541 |
| BIO18 - Precipitation of Warmest Quarter | 0.072641 | 0.165595 |
| BIO19 - Precipitation of Coldest Quarter | 0.158761 | 0.333792 |
